# Supplementary material for: Hybrid Models and Biological Model Reduction with PyDSTool
Source: PLoS Comput Biol. 2012 Aug 9;8(8):e1002628. doi: 10.1371/journal.pcbi.1002628 (PMC3415397; doi:10.1371/journal.pcbi.1002628)
Supplement: Text S4 — Complete source code for the PyDSTool package (version 0.88.120504). Includes API documentation and help files linking to web pages. This file is identical to the current public release on Sourceforge.net. (ZIP) [file pcbi.1002628.s004.zip › PyDSTool/html/PyDSTool.Toolbox.optimizers.tests.test_rosenbrock-pysrc.html]

xml version="1.0" encoding="ascii"?


PyDSTool.Toolbox.optimizers.tests.test\_rosenbrock


| Home | Trees | Indices | Help | PyDSTool |
| --- | --- | --- | --- | --- |

Package PyDSTool ::
Package Toolbox ::
Package optimizers ::
Package tests ::
Module test\_rosenbrock

[hide private]

[frames] | no frames]

# Source Code for Module PyDSTool.Toolbox.optimizers.tests.test\_rosenbrock

```
 1  #!/usr/bin/env python 
 2   
 3  # Matthieu Brucher 
 4  # Last Change : 2007-08-24 10:59 
 5   
 6  """ 
 7  Class defining the Rosenbrock function 
 8  """ 
 9   
10  import numpy 
11  from numpy.testing import * 
12  set_package_path() 
13  from optimizers import criterion, step, optimizer, line_search 
14  restore_path() 
15   


16 -class Rosenbrock:


17    """ 
18    The Rosenbrock function 
19    """ 


20 -  def __init__(self, dimension):


21      """ 
22      Constructor 
23      """ 
24      self.dimension = dimension

25   


26 -  def __call__(self, x):


27      """ 
28      Get the value of the Rosenbrock function at a specific point 
29      """ 
30      return numpy.sum(100.0 * (x[1:] - x[:-1]**2.0)**2.0 + (1. - x[:-1])**2.0)

31   


32 -  def gradient(self, x):


33      """ 
34      Evaluates the gradient of the function 
35      """ 
36      xm = x[1:-1] 
37      xm_m1 = x[:-2] 
38      xm_p1 = x[2:] 
39      der = numpy.zeros(x.shape, x.dtype) 
40      der[1:-1] = 200. * (xm - xm_m1**2.) - 400. * (xm_p1 - xm**2.) * xm - 2. * (1. - xm) 
41      der[0] = -400. * x[0] * (x[1] - x[0]**2.) - 2. * (1. - x[0]) 
42      der[-1] = 200. * (x[-1] - x[-2]**2.) 
43      return der

44   


45 -  def hessian(self, x):


46      """ 
47      Evaluates the gradient of the function 
48      """ 
49      H = numpy.diag(-400. * x[:-1], 1) - numpy.diag(400. * x[:-1],-1) 
50      diagonal = numpy.zeros(len(x), x.dtype) 
51      diagonal[0] = 1200. * x[0]**2. - 400. * x[1] + 2. 
52      diagonal[-1] = 200. 
53      diagonal[1:-1] = 202. + 1200. * x[1:-1]**2. - 400. * x[2:] 
54      H += numpy.diag(diagonal) 
55      return H

56   


57 -class test_Rosenbrock(NumpyTestCase):


58    """ 
59    Global test class with the Rosenbrock function 
60    """ 


61 -  def check_simple_gradient_monotony(self):


62      startPoint = numpy.empty(2, numpy.float) 
63      startPoint[0] = -1.01 
64      startPoint[-1] = 1.01 
65      optimi = optimizer.StandardOptimizer(function = Rosenbrock(2), step = step.GradientStep(), criterion = criterion.OrComposition(criterion.MonotonyCriterion(0.00001), criterion.IterationCriterion(10000)), x0 = startPoint, line_search = line_search.SimpleLineSearch(alpha_step = 0.001)) 
66      assert_almost_equal(optimi.optimize(), numpy.ones(2, numpy.float), decimal=1)

67   


68 -  def check_simple_gradient_relative(self):


69      startPoint = numpy.empty(2, numpy.float) 
70      startPoint[0] = -1.01 
71      startPoint[-1] = 1.01 
72      optimi = optimizer.StandardOptimizer(function = Rosenbrock(2), step = step.GradientStep(), criterion = criterion.RelativeValueCriterion(0.00001), x0 = startPoint, line_search = line_search.SimpleLineSearch(alpha_step = 0.001)) 
73      assert_almost_equal(optimi.optimize(), numpy.ones(2, numpy.float), decimal=1)

74   


75 -  def check_simple_newton_relative(self):


76      startPoint = numpy.empty(2, numpy.float) 
77      startPoint[0] = -1.01 
78      startPoint[-1] = 1.01 
79      optimi = optimizer.StandardOptimizer(function = Rosenbrock(2), step = step.NewtonStep(), criterion = criterion.RelativeValueCriterion(0.00001), x0 = startPoint, line_search = line_search.SimpleLineSearch()) 
80      assert_almost_equal(optimi.optimize(), numpy.ones(2, numpy.float))

81   


82 -  def check_wpr_cwgradient(self):


83      startPoint = numpy.empty(2, numpy.float) 
84      startPoint[0] = -1.01 
85      startPoint[-1] = 1.01 
86      optimi = optimizer.StandardOptimizer(function = Rosenbrock(2), step = step.CWConjugateGradientStep(), criterion = criterion.criterion(iterations_max = 1000, ftol = 0.00000001, gtol = 0.0001), x0 = startPoint, line_search = line_search.WolfePowellRule()) 
87      assert_array_almost_equal(optimi.optimize(), numpy.ones(2, numpy.float))

88   


89 -  def check_swpr_dygradient(self):


90      startPoint = numpy.empty(2, numpy.float) 
91      startPoint[0] = -1.01 
92      startPoint[-1] = 1.01 
93      optimi = optimizer.StandardOptimizer(function = Rosenbrock(2), step = step.DYConjugateGradientStep(), criterion = criterion.criterion(iterations_max = 1000, ftol = 0.00000001, gtol = 0.0001), x0 = startPoint, line_search = line_search.StrongWolfePowellRule()) 
94      assert_array_almost_equal(optimi.optimize(), numpy.ones(2, numpy.float), decimal = 4)

95   
96  if __name__ == "__main__": 
97    NumpyTest().run() 
98
```

  


| Home | Trees | Indices | Help | PyDSTool |
| --- | --- | --- | --- | --- |

|  |  |
| --- | --- |
| Generated by Epydoc 3.0.1 on Fri May 4 15:24:20 2012 | http://epydoc.sourceforge.net |
